# 

**Published:** 1891-11-28

**Authors:** 


					for: INFANTS
And INVALIDS
1 rt n a nffiljfj
iMMyjjjj '"Mflj
ifffjlffrnn
Clascoiy Western Infirmary*"^
S'/Thomas s Hospital
ahb NORWICH HOSPt.
? WITH extra NURSING SUPPLEMENT.
No. 270. Vol. XI.] Saturday, November 28^1i, 1891

				

